# Integrating data and knowledge to identify functional modules of genes: a multilayer approach

**DOI:** 10.1186/s12859-019-2800-y

**Published:** 2019-05-02

**Authors:** Lifan Liang, Vicky Chen, Kunju Zhu, Xiaonan Fan, Xinghua Lu, Songjian Lu

**Affiliations:** 1 Department of Biomedical Informatics, University of Pittsburgh, Pittsburgh, PA USA; 2 Frederick National Laboratory for Cancer Research, Leidos Biomedical Research, Inc, Frederick, USA; 3 Key Laboratory of Information Fusion Technology of Ministry of Education, School of Automation, Northwestern Polytechnical University, Xi’an, 710072 Shanxi China; 4 Clinical Medicine Research Institute, Jinan University, Guangzhou, 51063 Guangdong China

**Keywords:** Protein-protein interaction, Graph clustering, Random walk, Multiplex, Topic modeling, Gene expression, Functional module, Protein complex

## Abstract

**Background:**

Characterizing the modular structure of cellular network is an important way to identify novel genes for targeted therapeutics. This is made possible by the rising of high-throughput technology. Unfortunately, computational methods to identify functional modules were limited by the data quality issues of high-throughput techniques. This study aims to integrate knowledge extracted from literature to further improve the accuracy of functional module identification.

**Results:**

Our new model and algorithm were applied to both yeast and human interactomes. Predicted functional modules have covered over 90% of the proteins in both organisms, while maintaining a comparable overall accuracy. We found that the combination of both mRNA expression information and biomedical knowledge greatly improved the performance of functional module identification, which is better than those only using protein interaction network weighted with transcriptomic data, literature knowledge, or simply unweighted protein interaction network. Our new algorithm also achieved better performance when comparing with some other well-known methods, especially in terms of the positive predictive value (PPV), which indicated the confidence of novel discovery.

**Conclusion:**

Higher PPV with the multiplex approach suggested that information from both sources has been effectively integrated to reduce false positive. With protein coverage higher than 90%, our algorithm is able to generate more novel biological hypothesis with higher confidence.

## Background

Understanding the mechanisms of pathway perturbations underlying complex human diseases remains a difficult problem, hindering the development of targeted therapeutics. Complex diseases involve many genes and molecules that interact within context-specific cellular networks, such as signaling networks, physical interaction networks, and co-expression networks [1]. For example, cancer was often viewed as the disruption of cellular signaling networks. Such complex networks are inherently modular [2], meaning that genes usually perform certain biological function in separate groups. Therefore, to investigate complicated cellular mechanism, it is necessary to characterize the modular structure of cellular networks.

A functional module is defined as a group of genes or their products which are related by one or more genetic or cellular interactions, e.g. co-regulation, co-expression or membership of a protein complex, of a metabolic or signaling pathway or of a cellular aggregate (e.g. chaperone, ribosome, protein transport facilitator) [3]. Since physical protein-protein interactions directly indicate the cooperation of gene products to drive a biological process, a variety of clustering methods were developed to identify functional modules from protein-protein interaction networks [4]. Zinman, et al. [5] have found that functional interactions that are part of functional modules are conserved at a much higher rates, further supporting the advantage of using protein interaction networks.

In the past decade, a vast amount of methods has been developed to identify functional modules in protein-protein interaction networks. As summarized by previous reviews [4], a majority of these algorithms can be categorized into: (1) density-based [6, 7], identifying densely connected groups of proteins; (2) partition-based [8], separate all sparsely connected nodes; (3) flow simulation-based [8–11], simulating a biological or functional flow; (4) core attachment-based [12–14], exploiting the core-attachment structure of protein relations. Recently, evolutionary algorithm [15, 16] has been adopted to avoid poor local minimum; and node embedding [17] have been used to transform the graph clustering problem into a conventional clustering problem. In addition, algorithms [18, 19] combining two or more approaches described above has emerged. Unfortunately, the computational methods for functional module identification are clearly limited by the poor quality of the underlying PPI data, which is noisy with high rates of false positive and false negative [20, 21]. However, the various approaches to identify functional modules have served as the foundation to inspire further improvement. In this study, we followed the flow simulation-based approach to capture the dynamics of multiplex networks.

Another popular approach is to identify functional modules from co-expression network. Unlike protein interaction networks, edges in co-expression networks indicate differential expression of two genes within the same sample or condition. It assumes that tightly interacting and functionally dependent proteins are co-expressed across most conditions. This assumption is a reliable heuristic for functional module identification, despite that co-expression is not direct evidence for functional relation. Studies had successfully identified stable functional modules from co-expression networks across species [22]. Therefore, expression status of co-expression functional modules should be highly related to activities or behavior of cells. Many biological studies have identified active functional modules related to certain diseases from co-expression networks [23, 24].

However, in the case of co-expression network, identifying functional modules at the appropriate granularity is a big challenge. As each experimental condition usually has perturbed multiple signaling pathways, differentially expressed genes in each condition usually correspond to multiple dysregulated biological processes [20]. This could result in predicted functional modules being a superset of several real functional modules.

In addition, high-throughput expression data also has its own data quality issues. For example, RNAseq data still suffered from technical issues, such as batch effects and contamination. Recent studies have developed different methods to improve accuracy of module identification by integrating co-expression network and protein interaction networks [3, 20, 25–30] or other heterogeneous data sources [31–33], while others integrated homogeneous data sources to improve confidence [34, 35]. However, data quality issues common in high-throughput data, especially the experimental aspects, remain unresolved.

Besides high-throughput data, decades of research efforts have obtained and validated vast amounts of biological knowledge through wet-lab experiments, which are valuable resources for further research. Such knowledge should contain much less errors compared to high-throughput data. A few studies have attempted to utilized the literature for functional module identification [36–39]. However, relying on literature alone may lead to findings biased towards well studied genes, providing less novel insights [40, 41].

Since high-throughput data is less biased towards well-known genes and literature has fewer data quality issues, integrating these two information sources seems promising [42]. Methods [17, 43, 44] have been proposed to integrate prior knowledge. However, some of the methods suffer two major issues: (1) identification is restricted in the scope of knowledge, resulting in knowledge bias unresolved; (2) adoption of strong prior knowledge, such as Gene Ontology, that may not be independent from the gold standard, leads to overly optimistic evaluation results.

To address the issues above, this study has followed a multilayer network approach for data integration. Multiplex is a natural way to represent interactions in a complex system from multiple perspectives [45]. By treating prior knowledge separately in one layer, the identification process is not confined by knowledge, but enhanced by knowledge. In addition, although it is common practice to aggregate multiplex into a single weighted network [46–48], research on multiplex suggested that important information can be lost during aggregation [49]. Thus, this study seeks to capture multiplex dynamics with random walk / diffusion theoretic.

Random walks on multiplex can induce congestion even when each single layer remains decongested [50]. Also, the fraction of nodes a random walker can travel has increased, owing to their resilience to uniformly random failures [51]. Thus, the dynamics of diffusion is able to capture the additional information brought by multiplex formation. In this study, we first computed the first k step visit probability of the nodes in the multiplex, which can be viewed as the uncompressed, exact solution for node embedding [52]. Then we identified modules on the probability matrix with an objective function, named isolation, that promotes both module density and minimum cut in terms of k-step connectivity.

Two major hypothesis were tested in this study: (1) gene-topic associations extracted from literature is able to reveal functional relations of genes and provide information complementary to high-throughput data; (2) integration of multiple information sources with multiplex approach can improve the accuracy of functional module identification.

## Result

We first identified differentially expressed genes from RNA expression data. Then we calculated topic-gene association from Pubmed titles and abstracts. These two types of data were used to calculate functional similarity among genes used as edge weights for protein interaction networks respectively. The two weighted PPI networks were further connected with the multiplex approach. Finally, we developed a clustering algorithm to identify functional modules with locally maximum isolation from the two-layer protein interaction network.

Our clustering algorithm on multiplex was compared with itself on single layer network to show the effectiveness to information integration. To further demonstrate its performance, a network integration algorithm named Similarity Network Fusion (SNF) [48] was also compared. Then the proposed algorithm was compared against other methods in terms of protein coverage and accuracy.

### Descriptive statistics

BioGrid curation of PPI for *Saccharomyces cerevisiae* contained 32,353 interactions among 4518 gene products. The transcriptomic profile of yeast perturbation experiments contained expression values of 5980 genes under 1525 knockout conditions. The topic-gene association matrix contained 216 topics and 5348 genes.

After network construction, the yeast interactome based on topic modeling had 4187 genes and 30,989 interactions; the yeast interactome based on transcriptomic profiles contained 4179 genes and 30,887 interactions; the interactome based on the combination of the transcriptomic interactome and the topic-gene associations contained 8302 genes and 65,793 interactions.

The protein interaction network contained 10,945 nodes and 56,471 edges. The transcriptomic profile of breast cancer patients in TCGA contained 1218 samples and 20,252 genes. The topic-gene association matrix contained 209 topics and 16,712 genes.

After network construction, the human interactome based on transcriptomic profiles contained 10,029 genes and 49,909 edges. The human interactome based on topic modeling contained 10,368 genes and 48,806 edges. The combined interactome contained 19,266 genes and 212,292 edges.

### Single-layer versus multiplex

**Fig. 1:**
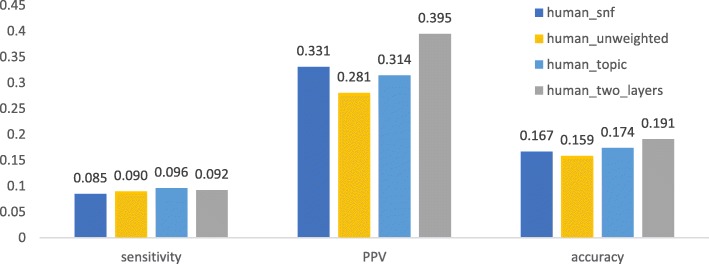
Performance of isolation clustering on three different human interactomes, using Gene Ontology as gold standard

**Fig. 2:**
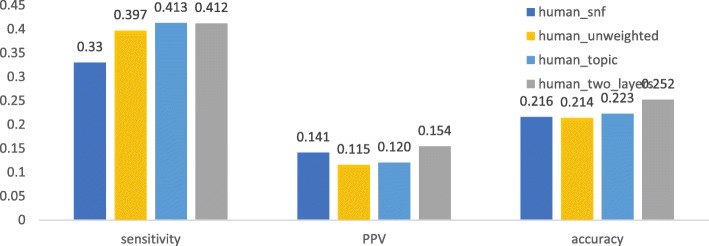
Performance of isolation clustering on three different human interactomes, using CORUM as gold standard

**Fig 3.**
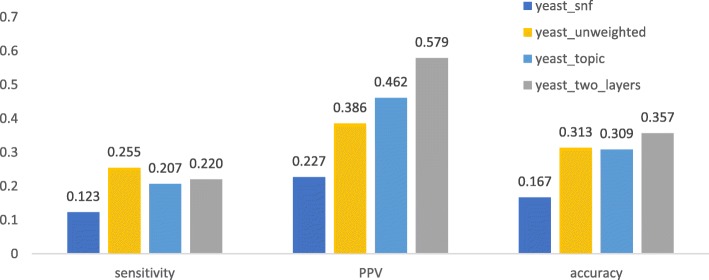
Performance of isolation clustering on three different yeast interactomes, using Gene Ontology as gold standard

**Fig. 4:**
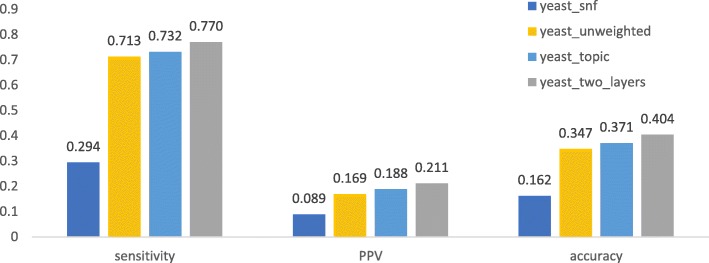
Performance of isolation clustering on three different human interactomes, using CYC2008 as gold standard

We first checked if a method using both knowledge and expression data can obtain better performance than those using only protein interaction networks or combined with topic associaion. As shown in Fig. 1, 2, 3, 4, after being weighted by topic association (“human_topic” and “yeast_topic” in the legend), sensitivity, PPV and accuracy have been improved improved across different datasets and different gold standards. It was shown that topic-association data provided additional information about functional relations among genes.

After integrating the interactomes weighted by topic association and gene co-expression (“human_two_layers” and “yeast_two_layers” in Figs. 1, 2, 3, 4), PPV was further improved while sensitivity decreased slightly. This suggests our algorithm tends to identify clusters with less false positives, at the cost of inducing a few false negatives. Overall, accuracy increased with the multiplex approach.

The performance of the network fusion approach (“human_snf” and “yeast_snf” in Figs. 1, 2, 3, 4) seems to differ in different datasets. In the case of the human interactome, SNF has increased PPV and decreased sensitivity, which is similar with our method, though the overall performance gain is not obvious. For the yeast interctome, SNF yielded a performance worse than the single layer clustering in terms of sensitivity, PPV and accuracy. The reason could be that the iterative matrix computation procedure of SNF is more likely to return an almost uniform distribution of edge weights if the network density is high.

### Comparison with other methods

We then compared our clustering method with some other well-known methods in terms of solution sizes, protein coverage, and accuracy. All the clusters with less than 3 proteins or larger than 200 proteins were removed. As shown in Tables 1 and 2, the distribution of cluster size for our method (isolation) is more skewed towards size 3–10. For the species of yeast, CYC2008 has over 83.3% of proteins with size less than 10, while the percentage of MCL, Infomap, Isolation was 73.8, 64.4, and 92.3% respectively. For the species of human, 89.5% of proteins complexes in CORUM contain less than or equal to 10 gene products, while 88.9% of functional modules generated by Isolation has such small size. Assuming that this distribution of CORUM and CYC2008 represents the true distribution of protein complexes, it indicated that the modular structure characterized by Isolation clustering was similar with that within real cells.

**Table 1:** The distribution of cluster size by different methods on yeast interactomes. The rightmost column is the gold standard used in this study

Size	MCL	Walktrap	Infomap	MCODE	ClusterOne	NCMine	Isolation	CYC2008
3–10	342	158	275	135	426	1096	995	198
11–50	107	44	140	21	86	612	82	36
51–100	13	5	7	11	10	23	1	2
100–200	0	5	5	2	4	0	0	0
> 200	1	0	0	0	0	0	0	0

**Table 2:** The distribution of cluster size by different methods on human interactomes. The rightmost column is the gold standard used in this study

Size	MCL	Walktrap	Infomap	MCODE	ClusterOne	NCMine	Isolation	CORUM
3–10	1008	323	506	319	1322	1943	2131	1562
11–50	353	83	241	47	108	253	260	176
51–100	15	13	16	8	0	0	4	5
100–200	0	3	3	1	0	0	1	2
> 200	0	0	0	3	0	0	0	0

### Protein coverage rates

**Fig. 5:**
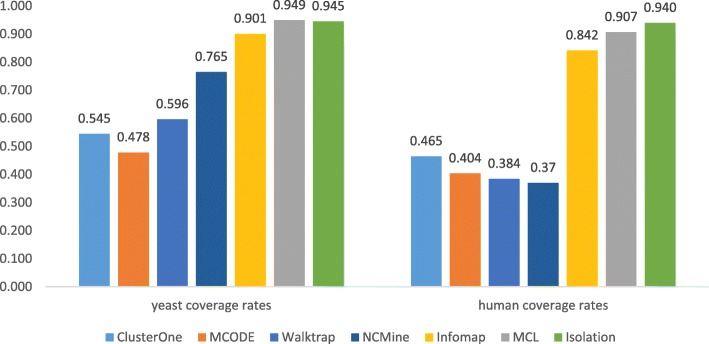
In clustering for both yeast and human interactomes, clustering based on random walks has covered most proteins, while density-based clustering discarded around half the proteins

As shown in Fig. 5, clusters generated by ClusterOne, MCODE, and Walktrap can only cover around half of the interactome. MCL, Infomap, and Isolation had covered over 90% of the interactome. Significantly higher coverages indicated that clustering methods based on random walks (i.e. MCL, Infomap, and Isolation) may provide more information about novel proteins so as to generate more biological insights. In the next section, only MCL, Infomap, and Isolation were compared against each other to in terms of accuracy.

### Geometric accuracy

**Fig. 6:**
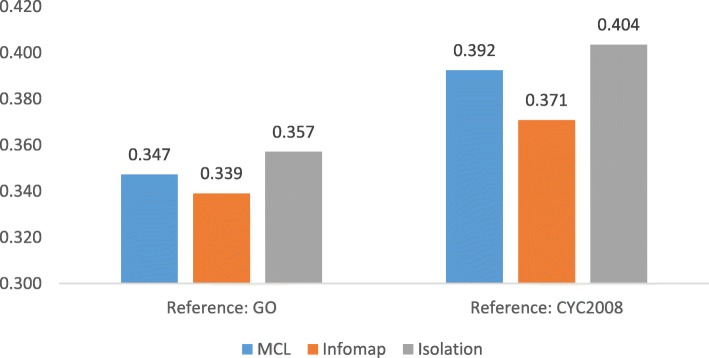
Comparison of geometric accuracy of MCL, Infomap, and isolation on yeast interactomes

**Fig. 7:**
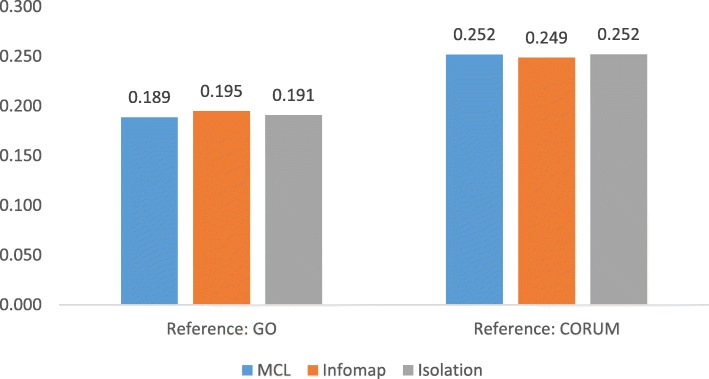
Comparison of geometric accuracy of MCL, Infomap, and isolation on human interactomes

As shown in Figs. 6 and 7, Isolation has outperformed MCL and Infomap in yeast interactome in terms of geometric accuracy. The accuracy by our method is slightly higher than other methods. However, in the case of human interactomes, these three methods yielded very similar performance in every aspect.

### Examples of clusters

**Fig. 8:**
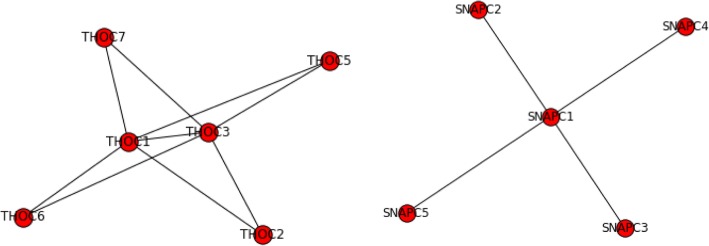
The two predicted complexes perfectly matched to CORUM complexes. On the left is matched to hTREX84 complex. On the right is matched to SNAPc complex

**Fig. 9:**
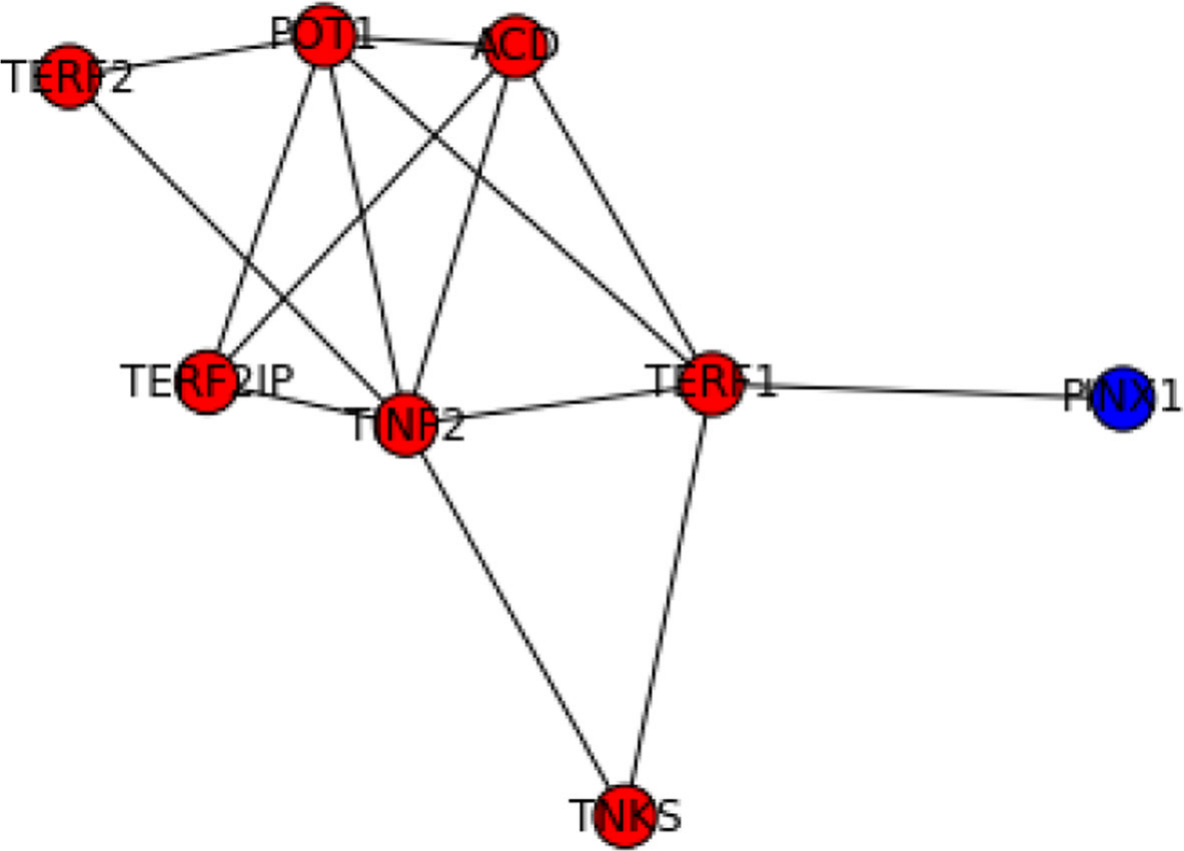
Predicted complex matched to telomere-associated protein complex and TRF-Rap1 complex I, 2MD. Blue nodes were genes predicted but absent in the gold standard

Our clustering results have found many overlaps with known complexes. Two of them were perfect matches (Fig. 8). For some genes misclassified to a complex, we are able to identify close functional relations from literature. For example, our methods had grouped PINX1 with TRF-Rap1 complex I (Fig. 9). Although PINX1 is not part of the complex, it is well studied that PINX1 can mediate TRF1 (or TERF1) and TERT accumulation in nucleus and enhances TERF1 binding to telomeres [53, 54], thus affecting the function of the complex.

**Fig. 10:**
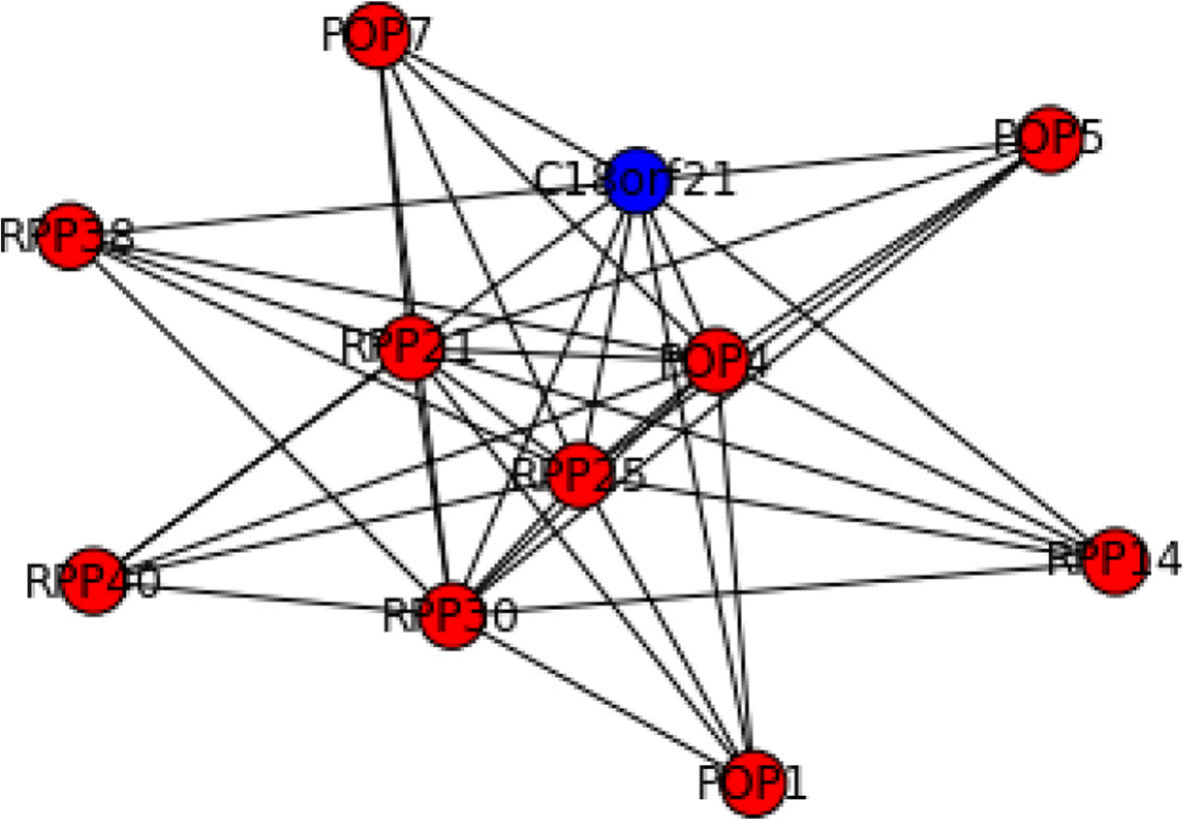
Predicted complex matched to Rnase/Mrp complex. Blue nodes were genes predicted but absent in the gold standard

**Fig. 11:**
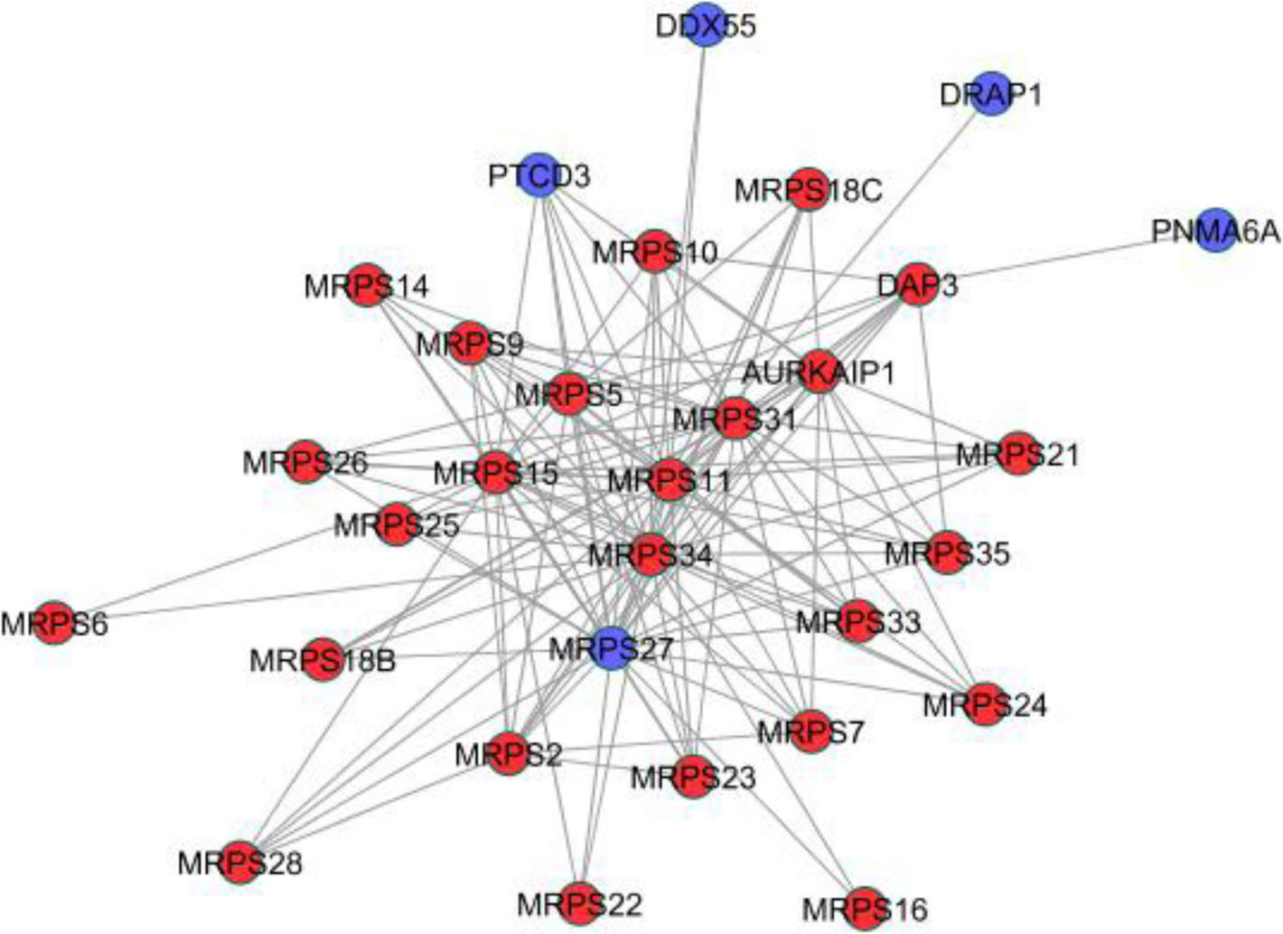
Predicted complex matched to 39S ribosomal subunit, mitochondrial. Blue nodes were genes predicted but absent in the gold standard

Furthermore, “misclassified” genes without direct evidence supports may be more interesting since they could provide new insights for current knowledge. For example, C18orf21 was grouped with Rnase/Mrp complex by our method (Fig. 10). Several studies have found genetic association between variants in C18orf21 and phenotypes of human. Besides the high-throughput data (BioPlex [55]) used in this study, no further experiments have been conducted to investigate the functions of C18orf21. Our results suggested that C18orf21 could function through regulating Rnase/Mrp complex. Another example was shown in Fig. 11, where PNMA6A, DRAP1, PTCD3, AURKAIP1, and DDX55 were grouped with the 28S ribosomal subunit. Through literature we found that these misclassified genes, except PNMA6A, have significant impact on mitochondrial ribosome though detailed mechanisms are not clear [56–58].

## Discussion

As illustrated in the result section, isolation clustering tends to identify isolated regions supported by both layers in the multiplex. Such tendency reduces false positives while inducing more false negatives. As shown in results, our new clustering algorithm, Isolation, has achieved better, or at least comparable, performance with other well-known clustering algorithms based on random walk. Particularly, subnetworks with locally maximal isolation exhibited higher confidence of being true positive when compared with MCL and Infomap. When compared with clustering algorithms such as ClusterOne, our algorithm has covered over 90% of proteins while density-based clustering can only cover around 50%. This leads to higher PPV from density-based clustering algorithms.

In addition, PPV is more important than sensitivity in terms of the confidence of true discovery. PPV of 1 indicates that the predicted module is a subset of a certain functional group in the gold standard, which means that every gene within the predicted module is related. On the other hand, sensitivity of 1 means a certain real functional module is a subset of a predicted module, which doesn’t validate other functional relationships among the predicted module. Thus when end users try to identify genes functionally relevant with a specific gene, it is natural to focus more on positive predictive value or precision rather than composite scores or sensitivity used by most methodological evaluations. From this perspective, our integrative approach provides more practical values.

However, since the method is trading sensitivity for PPV, it could be problematic when data is more prevalent with false negative than false positive. In most cases, this means our algorithm is more suitable for dense networks. Users of our method should analyze the sparsity of the network before conducting the algorithm.

Selected examples in the result section have shown that false positive genes could be functionally related in a way other that protein complexes. This illustrated one fundamental limitation for functional module identification and its evaluation. Biological experiments should be conducted to further verify the predicted modules.

This study also demonstrated that topic modeling of biomedical literature is an effective complementary source of information. Knowledge validated and curated in the form of literature are generally more reliable than high-throughput data. By integrating knowledge into the functional module identification process, false positives caused by data quality issues can be reduced. Thus, functional modules are identified with higher confidence. However, topic modeling of biomedical literature is not an easy task. The nHDP model used in this study took roughly 7 days to generate the topic mixtures. Future research may consider alternative information sources for integration.

## Conclusion

In this paper, we have proposed a multiplex approach to integrate high-throughput data and literature for functional module identification and developed a clustering method that can utilize the topology based on random walk. Results showed that our algorithm is able to generate more novel biological hypothesis with higher confidence.

## Methods

### Topic modeling of genes

The title and abstract information of biomedical articles were downloaded from Pubmed. First, by treating each gene as a document, tf-idf scores were calculated to identify words most pertinent to a certain gene. To filter the documents, words with tf-idf scores lower than 167 were removed; and the vocabulary was restricted to 13,000. Second, a word vector was then created for each gene by going through its list of 200 words with the highest tf-idf scores and including only the ones that occur in the vocabulary. For each cancer sample, word vectors for its differentially expressed genes were combined. nHDP [59] was used to identify the latent topics in the set of combined word vectors.

Topic-document associations and topic-word associations generated from nHDP were further utilized to calculate the gene-topic association scores used in this study. Association strength between a certain gene *g* and a certain topic *t* was calculated by the total sum of products of: (1) a specific word w’s count in *g*’s word vector, (2) *t*’s probability in document d, (3) the word w’s probability in t. For detailed description of this section, please see steps A-E in [37, 60].

### Similarity measure

Functional similarity among genes was calculated with topic-gene association matrix and transcriptomic profiles respectively.

For the topic-gene association matrix, association scores less than one were set zero. Measure of similarity was computed based on Simrank [61]:

1 $$ {T}_i={c}_1\left({S}^T{G}_i\mathrm{S}\right) $$

2 $$ {G}_i={c}_2\left({S}^T{T}_{i-1}S\right) $$

where S was a *g by n* matrix containing the association score between n topics and g genes, *G*_*i*_ was the g by g matrix containing the similarity among genes in the *i*th iteration, *T*_*i*_ was the n by n matrix containing the similarity among topics in the *i*th iteration, and *c*_1_ and *c*_2_ were the hyper-parameters controlling the impact of later iterations. In this study, both *c*_1_ and *c*_2_ were set to 0.8. The eq. (1) and (2) were iterated until T and G reach convergence. Note that only the similarity matrix G was used in the next section.

For the transcriptomic profile data, expression values were dichotomized. Genes expressions higher or lower than 95% interval of the distribution was encoded as one, otherwise zero. Cosine similarity was used to compute the similarity among genes, which is:

3 $$ {sim}_{ij}=\frac{{\mathit{\exp}}_i\bullet {\mathit{\exp}}_j}{\sqrt{\left\Vert {\mathit{\exp}}_i\right\Vert \bullet \left\Vert {\mathit{\exp}}_j\right\Vert }} $$

where *exp*_*i*_ was the vector of expression values of the ith gene across all the experiments, ‖*exp*_*i*_‖ is the L2 norm of that vector.

### Network construction

#### Computation of similarity matrix

Protein-protein interaction (PPI) networks were used as the base network. The similarity measures computed in the last section were used as the edge weights for these PPI networks. Thus, the topic-based interactome consisted of the topology of a PPI network with edge weights from topic-gene association matrix; and the expression-based interactome consisted of the topology of a PPI network with edge weights from transcriptomic profile data. For PPI curated in BioGrid for yeast, we only selected interactions supported by at least two studies.

**Fig 12.**
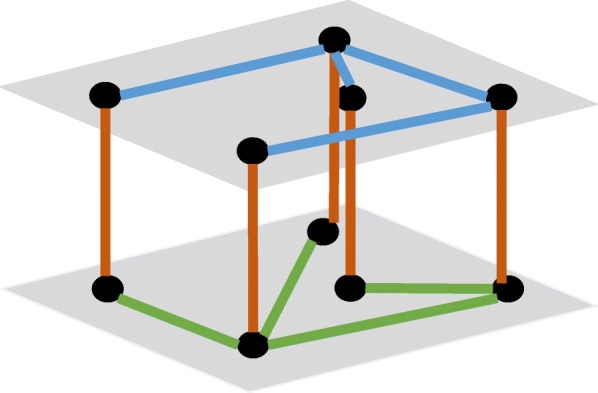
Illustration of the combined interactome, brown edges were artificial edges added to connect these two layers

These two interactomes were further combined into one network by treating each interactome as a layer and connecting the same gene across different layers, as demonstrated in Fig. 12.

#### Network integration

For all the networks described above, self-loops were removed. Edges with zero similarity and nodes with zero weighted degree were removed. The combined network is represented by supra-adjacency matrix [62]:

$$ A=\left[\begin{array}{cc}{A}_1& {I}_N\\ {}{I}_N& {A}_2\end{array}\right] $$

where *A*_*i*_ is the adjacency matrix for the ith layer, *I*_*N*_ is an N by N identify matrix, N is the number of nodes in a single layer.

### Clustering algorithm

The algorithm developed in this study consists of two steps: (1) transform the adjacency matrix into a matrix representing k-step walks visiting probability; (2) enumerate each node to identify clusters with locally optimal isolation.

#### Network transformation

With the network constructed from previous steps, the Markov transition matrix, M, should be computed next, which is:

4 $$ {M}_{ij}={A}_{ij}/{A}_{i.} $$

where *A*_*i*._ is the sum of the ith row of A.

From M, we further computed a matrix C, where *C*_*ij*_ is the probability that node j is visited if a walk of K steps start from node i. In this study, K is always set to 10. Since *C*_*ij*_ is complementary to the probability that node j never show up in the path, it can be computed as:

5 $$ {C}_{ij}=1-{{\mathbf{1}}_i}^{\boldsymbol{T}}\ {\left(M{I}_{-j}\right)}^K\mathbf{1} $$

where **1**_*i*_ is the vector with only the ith element as one, others zero, *I*_−*j*_ is an identity matrix with the jth diagonal value zero, **1** is the vector of 1.

As the vectorization of the operation above, the matrix C can be computed by the procedure below:

#### Objective function

Let us denote *t*_*ij*_ as the number of times node j is present in the path started from node i, then *t*_*ij*_ is sampled from a Bernoulli distribution with probability *C*_*ij*_. Thus, *C*_*ij*_ can also be viewed as the expected number of times node j is present if a k-step walk is started from node i, which is:

6 $$ {C}_{ij}=\mathit{\Pr}\left({t}_{ij}=1\right)=E\left({t}_{ij}\right) $$

We further denote R as a subset of nodes, and *t*_*iR*_ as the total number of nodes of R present in the walk:

7 $$ {t}_{iR}={\sum}_{j\in R}{t}_{ij} $$

According to linearity of expectation, we can derive that:

8 $$ \mathrm{E}\left({t}_{iR}\right)={\sum}_{j\in R}{C}_{ij} $$

We can further generalize the equation by denoting *t*_*QR*_ as the total number of nodes in R present in a walk started from a node in Q. A walk is started from a node i in R for *W*_*i*_ times. From the law of total expectation, we can derive that:

9 $$ E\left({t}_{QR}\right)={\sum}_{i\in R}{\sum}_{j\in Q}{W}_j{C}_{ij} $$

Assuming *W*_*j*_ =1 for every j, we developed two measures to capture the degree of isolation of a subset R. One is retention:

10 $$ retention=\frac{E\left({t}_{RR}\right)}{E\left({t}_{RG}\right)}=\frac{\sum_{i\in R}{\sum}_{j\in R}{C}_{ij}}{\sum_{i\in R}{\sum}_{j\in G}{C}_{ij}} $$

where G is the subset for all the nodes within the graph, *t*_*RR*_ is the expected number of nodes of R visited in the k-step walks started from each node in R once, *t*_*RG*_ is the expected total number of nodes of G visited in the k-step walks started from each node in R once. The higher retention, the more likely walkers started in R will stay in R.

The other is:

11 $$ exclusivity=\frac{E\left({t}_{RR}\right)}{E\left({t}_{GR}\right)}=\frac{\sum_{i\in R}{\sum}_{j\in R}{C}_{ij}}{\sum_{i\in G}{\sum}_{j\in R}{C}_{ij}} $$

where *t*_*GR*_ is the expected total number of nodes of R visited in the k-step walks started from all the nodes in G once. The higher exclusivity, the less likely walkers outside R will get in.

**Fig. 13:**
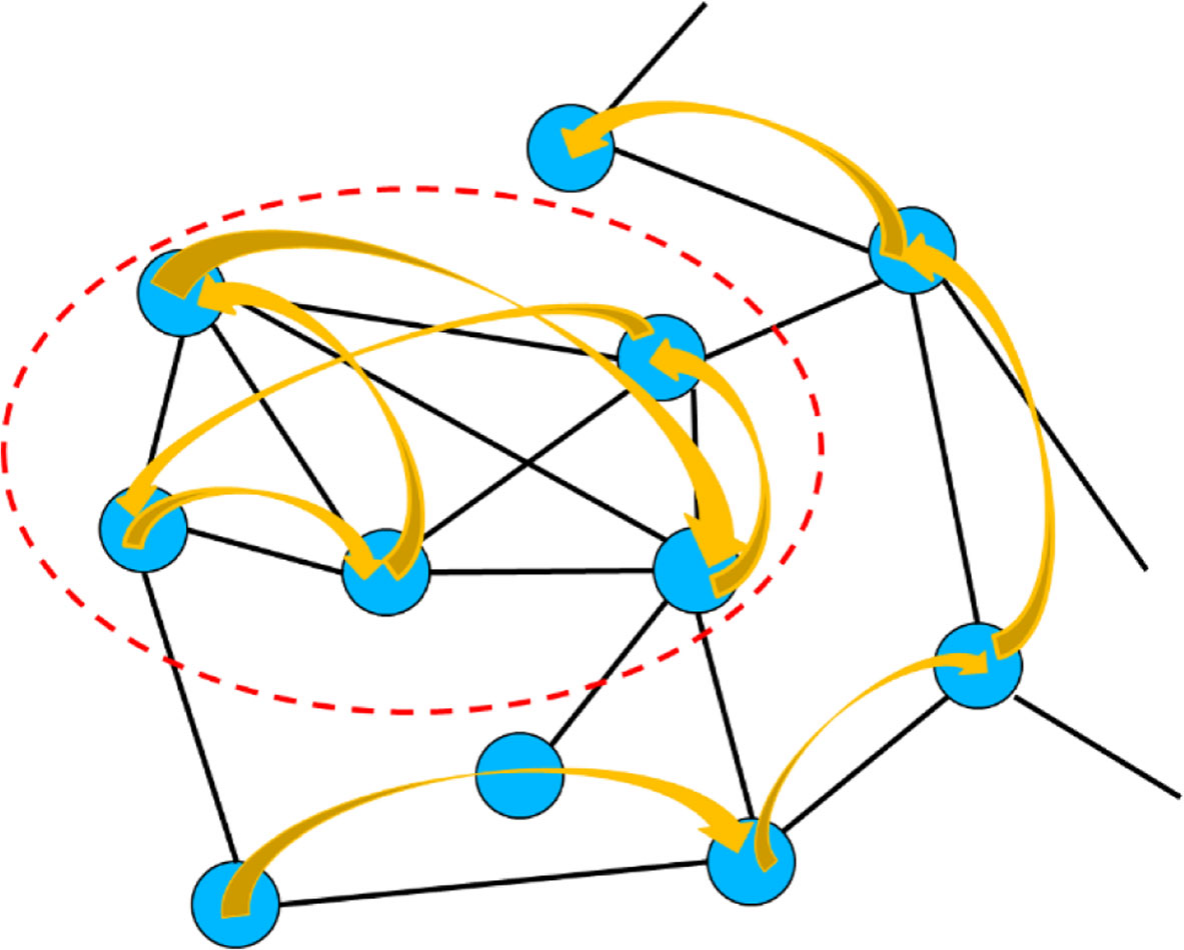
Illustration of the intuition of the objective function. Nodes within the red dotted circle would be a region with high isolation since walkers inside are likely to stay within and walkers outside are unlikely to get in

Combining these two measures, the objective function, named isolation in this study, is (Fig. 13):

12 $$ {isolation}_{RR}=\frac{E\left({t}_{RR}\right)}{E\left({t}_{RG}\right)+E\left({t}_{GR}\right)}=\frac{\sum_{i\in R}{\sum}_{j\in R}{C}_{ij}}{\sum_{i\in R}{\sum}_{j\in G}{C}_{ij}+{\sum}_{i\in G}{\sum}_{j\in R}{C}_{ij}} $$

#### Optimization procedures

To identify clusters with maximal isolation, we adopted a greedy approach iterating between two phases.

One is expansion. In the expansion phase, isolation is calculated for each individual node outside the cluster:

13 $$ {isolation}_{iR}=\frac{\sum_{j\in R}{C}_{ij}+{\sum}_{j\in R}{C}_{ji}}{\sum_{j\in G}{C}_{ij}+{\sum}_{j\in G}{C}_{ji}} $$

Top 10 nodes with *isolation*_*iR*_ higher than the original cluster are added into the cluster.

The other is shrinking. In this phase, isolation is calculated for each individual node within the cluster. All the nodes with *isolation*_*iR*_ lower than original cluster are removed from the cluster.

The algorithm keeps iterating between expansion and shrinking until there is no more qualified nodes for expansion.

### Proof of convergence

For expansion, let us denote the set of qualified nodes as X and the resulting cluster as R’. For each node i within X, *isolation*_*iR*_ > *isolation*_*RR*_. In other words:

$$ \frac{\sum \limits_{i\in X}{\sum}_{j\in R}{C}_{ij}+\sum \limits_{i\in X}{\sum}_{j\in R}{C}_{ji}}{\sum_{i\in X}{\sum}_{j\in G}{C}_{ij}+{\sum}_{i\in G}{\sum}_{j\in X}{C}_{ij}}>\frac{E\left({t}_{RR}\right)}{E\left({t}_{RG}\right)+E\left({t}_{GR}\right)} $$

Thus:

$$ \frac{E\left({t}_{RR}\right)+\sum \limits_{i\in X}{\sum}_{j\in R}{C}_{ij}+\sum \limits_{i\in X}{\sum}_{j\in R}{C}_{ji}}{E\left({t}_{RG}\right)+E\left({t}_{GR}\right)+{\sum}_{i\in X}{\sum}_{j\in G}{C}_{ij}+{\sum}_{i\in G}{\sum}_{j\in X}{C}_{ij}}>\frac{E\left({t}_{RR}\right)}{E\left({t}_{RG}\right)+E\left({t}_{GR}\right)} $$

On the other hand,

$$ {isolation}_{R\prime R\prime }=\frac{E\left({t}_{RR}\right)+\sum \limits_{i\in X}{\sum}_{j\in R}{C}_{ij}+\sum \limits_{i\in X}{\sum}_{j\in R}{C}_{ji}+E\left({t}_{XX}\right)}{E\left({t}_{RG}\right)+E\left({t}_{GR}\right)+{\sum}_{i\in X}{\sum}_{j\in G}{C}_{ij}+{\sum}_{i\in G}{\sum}_{j\in X}{C}_{ij}}>\frac{E\left({t}_{RR}\right)+\sum \limits_{i\in X}{\sum}_{j\in R}{C}_{ij}+\sum \limits_{i\in X}{\sum}_{j\in R}{C}_{ji}}{E\left({t}_{RG}\right)+E\left({t}_{GR}\right)+{\sum}_{i\in X}{\sum}_{j\in G}{C}_{ij}+{\sum}_{i\in G}{\sum}_{j\in X}{C}_{ij}} $$

Hence *isolation*_*R* ′ *R*′_ > *isolation*_*RR*_ after expansion.

Similarly, increase of isolation after shrinking can be proved. Thus, our objective function, isolation, is always increasing during iterations and convergence is guaranteed.

#### Merging overlapped clusters

Highly overlapping clusters are likely to exist for this method. Additionally, for integrated networks, duplicate gene IDs in the same cluster need to be removed. Therefore, overlapping among clusters were evaluated by Jaccard coefficients:

14 $$ overlap\left({C}_i,{C}_{\mathrm{j}}\right)=\frac{\left|{C}_i\cap {C}_j\right|}{\left|{C}_i\cup {C}_j\right|} $$

where *C*_*i*_ was the *i*th cluster, |*C*_*i*_| was the number of genes in *C*_*i*_. *C*_*i*_ ∩ *C*_*j*_ was the intersection of *C*_*i*_ and *C*_*j*_, and *C*_*i*_ ∩ *C*_*j*_ is the union of *C*_*i*_ and *C*_*j*_. A graph with clusters as nodes was constructed. There is an edge between cluster i and j if *overlap*(*C*_*i*_, *C*_*j*_) > 0.8. Collections of highly overlapping clusters is identified as a connected component of the graph. Union and intersection of all the highly overlapping clusters is computed and added into the collection as new clusters. For each collection, the cluster with highest isolation will remain while all the others will be removed.

### Evaluation

#### Metrics

Three sets of measures were adopted in this study to evaluate the clustering performance: (1) Geometric accuracy [21], PPV [21], sensitivity [21]; (2) F measure [63], precision [63], and recall [63]; (3) protein coverage rates, which is the number of unique proteins included by the clustering methods over the total number of proteins of the interactome.

### Precision, recall and F-measure

Let P denote the sets of complexes predicted by a computational method and B the real ones in the gold standard. The neighborhood affinity score NA(p, b) between the element p in P, *V*_*p*_, and the element b in B is defined as:

$$ NA\left(p,b\right)=\frac{{\left|{V}_p\cap {V}_b\right|}^2}{\mid {V}_p\mid \times \mid {V}_b\mid } $$

where *V*_*p*_ is the set of vertexes in the predicted subnetwork p. When NA(p,b) > 0.2, we say p matches b, or vice versa. Thus precision and recall can be defined as:

$$ Precision=\frac{N_{cp}}{\mid P\mid } $$

$$ Recall=\frac{N_{cb}}{\left|B\right|} $$

where *N*_*cp*_ is the number of elements in P that matches at least one element in B and *N*_*cb*_ the number of elements in B that matches at least one element in P. F-measure, or F1, can then be defined as:

$$ F=\frac{2\times Precision\times Recall}{Precision+ Recall} $$

### Sensitivity, positive predictive value (PPV) and geometric accuracy

With the notations above, let’s denote the overlap between p and b as:

$$ {T}_{pb}=\left|{V}_p\cap {V}_b\right| $$

Sensitivity and PPV are then defined as:

$$ Sensitivity=\frac{\sum_b^B\underset{p}{\max}\left\{{T}_{pb}\right\}}{\sum_b^B\mid {V}_b\mid } $$

$$ PPV=\frac{\sum_p^P\underset{b}{\max}\left\{{T}_{pb}\right\}}{\sum_p^P\mid {V}_p\mid } $$

As a summary metric, geometric accuracy, or simply accuracy, is defined as:

$$ Accuracy=\sqrt{Sensitivity\times PPV} $$

### Protein coverage

Denote *V*_*P*_ as:

$$ {V}_P= Union\left(\left\{{V}_p|p\in P\right\}\right) $$

Then protein coverage can be defined as:

$$ Coverage=\frac{\mid {V}_P\mid }{N} $$

where N is the total number of proteins in the interactome.

#### Experiment datasets

Three types of data were collected, including protein-protein interactions, transcriptomic profiles, and research paper titles and abstracts from NCBI. BioPlex 2.0 [55] was used as the protein interaction network. RNAseq data about 1097 breast cancer patients stored in TCGA [64] was used as the transcriptomic profile data. For the application on yeast interactome, protein interaction data was collected from BioGrid (version 3.4.162). Microarray datasets about systematic perturbation in yeast [25, 65] was also used in this study. We download over one million paper titles and abstracts from NCBI on March 23rd, 2016 for yeast and on September 25th, 2017 for human.

#### Gold standard

Gene Ontology (GO) and manually curated database of protein complexes were used as gold standard. The GO annotation for *Homo sapiens* was downloaded from Gene Ontology consortium. This file annotated 19,473 gene products and was submitted on September 26, 2017. The GO annotation file for *Saccharomyces cerevisiae* was downloaded from Saccharomyces Genome Database (SGD). This file annotated 6357 gene products with 4393 GO terms, 2104 of which are in the category of biological process. The list of 408 yeast protein complex was derived from CYC2008 [66], covering 1627 unique genes. Human protein complexes were downloaded from CORUM [67], with 2693 complexes and 4413 unique genes.

#### Control methods

We used Infomap [68], Markov Clustering algorithm (MCL), MCODE, Walktrap [69], NCMine [13] and ClusterOne [70] as the methods for comparison. These algorithms have been widely used to identify functional modules or protein complexes in protein interaction networks. We implemented MCL based on the work of Enright [9], used igraph to run Infomap and Walktrap, and used Cytoscape to run ClusterOne, NCMine and MCODE.

Since multiplex is applicable to most clustering methods based on random walk. Performance of MCL, Infomap, and Walktrap were evaluated based on their results on multiplex. For density-based clustering methods (i.e. MCODE, ClusterOne) and core-attachment approaches (i.e. NCMine), clustering results of the single layer with better performance were shown and compared.

In addition, we compared the multiplex approach with Similarity Network Fusion (SNF). The aggregated matrix generated by SNF is fed to the Isolation clustering algorithm. Our preliminary results showed that the network transformation step in the clustering procedure yields a uniform distribution of edges weights for most nodes. Therefore, the performance of SNF shown in the result section was generated by conducting merely the optimization step in the isolation algorithm.

## Abbreviations

GO: Gene Ontology
NCBI: National Center for Biotechnology Information
nHDP: Nested hierarchical dirichlet process
PPI: Protein-protein interactions
PPV: Positive predictive value
SNF: Similarity network fusion
TCGA: The Cancer Genome Atlas
